# Prediction of motif-mediated viral mimicry through the integration of host–pathogen interactions

**DOI:** 10.1007/s00203-024-03832-9

**Published:** 2024-02-09

**Authors:** Sobia Idrees, Keshav Raj Paudel, Philip M. Hansbro

**Affiliations:** 1 School of Biotechnology and Biomolecular Sciences, University of New South Wales, Sydney, NSW Australia; 2 Centre for Inflammation, School of Life Sciences, Faculty of Science, Centenary Institute and the University of Technology Sydney, Sydney, NSW Australia

**Keywords:** Virus-host interactions, Bioinformatics, Viral mimicry, Short linear motifs

## Abstract

One of the mechanisms viruses use in hijacking host cellular machinery is mimicking Short Linear Motifs (SLiMs) in host proteins to maintain their life cycle inside host cells. In the face of the escalating volume of virus-host protein–protein interactions (vhPPIs) documented in databases; the accurate prediction of molecular mimicry remains a formidable challenge due to the inherent degeneracy of SLiMs. Consequently, there is a pressing need for computational methodologies to predict new instances of viral mimicry. Our present study introduces a DMI-*de-novo* pipeline, revealing that vhPPIs catalogued in the VirHostNet3.0 database effectively capture domain-motif interactions (DMIs). Notably, both affinity purification coupled mass spectrometry and yeast two-hybrid assays emerged as good approaches for delineating DMIs. Furthermore, we have identified new vhPPIs mediated by SLiMs across different viruses. Importantly, the *de-novo* prediction strategy facilitated the recognition of several potential mimicry candidates implicated in the subversion of host cellular proteins. The insights gleaned from this research not only enhance our comprehension of the mechanisms by which viruses co-opt host cellular machinery but also pave the way for the development of novel therapeutic interventions.

**Supplementary Information:**

The online version contains supplementary material available at 10.1007/s00203-024-03832-9.

## Introduction

Viruses, as obligatory pathogens, depend on host cellular machinery for replication, establishing intricate interactions with host proteins throughout their life cycle. These interactions involve hijacking cellular components and countering host defences, posing challenges in the timely identification of targeted viral and host proteins (Rampersad and Tennant 2018; Sumbria et al. 2020; Bhutkar et al. 2022). Molecular mimicry by viruses, particularly through Short Linear Motifs (SLiMs) (Glavina et al. 2018; Venkatakrishnan et al. 2020; Idrees and Paudel 2023a; Idrees et al. 2023), has become an intriguing area of study, allowing viruses to effectively replicate, colonise host cells, and evade detection (Benedict et al. 2002; Finlay and McFadden 2006; Hraber et al. 2020; Goswami et al. 2023; Mihalič et al. 2023). In recent years, different computational studies have been conducted to study the host–pathogen interactions (Dyer et al. 2008). For example, Wadie et al. have leveraged viral motif mimicry to enhance the discovery of human linear motifs. This approach identified numerous putative linear motifs, with proteins engaged in motif-based interactions being more likely to be essential. Notably, these motif-based interactions are promising targets for addressing viral infections and associated diseases (Wadie et al. 2022). While computational studies on host–pathogen interactions have made significant strides, many have focused on specific pathogens (Barnes et al. 2016; Becerra et al. 2017). For instance, Castilla et al. utilized computational methods to explore molecular mimicry between the SARS-CoV-2 Spike protein and known epitopes, revealing key hotspots with potential autoimmune implications (Nunez-Castilla et al. 2022).

Despite this progress, the challenge remains in targeting Domain Motif Interactions (DMIs), known for their transient, complex, and promiscuous nature (Corbi-Verge and Kim 2016). The recent advancements in Protein–Protein Interaction (PPI) detection techniques, such as yeast two-hybrid (Y2H) and affinity purification coupled mass spectrometry (AP-MS), have provided large-scale virus-host PPI(vhPPI) data (de Chassey et al. 2014a). Y2H has been pivotal in 15 high-throughput screens for genome-wide viral interactomes, with initial genome-wide vhPPI screens for Hepatitis C Virus (de Chassey et al. 2008) and Epstein Barr virus (Calderwood et al. 2007). Y2H has been employed for targeted vhPPI identification, as exemplified by a study using ~ 12,000 human proteins and 10 influenza virus proteins (Shapira et al. 2009). Tandem affinity purification (TAP), a variation of AP-MS, is widely utilized for its efficacy in identifying numerous vhPPIs, boasting low contamination and a reduced rate of false positives (Pichlmair et al. 2012; Rozenblatt-Rosen et al. 2012). Approximately 30% of human proteins contain intrinsically disordered regions (IDRs) where SLiMs reside, showcasing the functional significance of these disordered regions (Idrees et al. 2023). SLiMs often mediate transient interactions because of their evolutionary plasticity and low-affinity interaction (Elkhaligy et al. 2021). Understanding SLiM-based interactions requires sophisticated methods, both experimental and computational. Tools like SLiMFinder (Edwards et al. 2007) and QSLiMFinder (Palopoli et al. 2015) contribute to *de-novo* SLiM discovery, helping to identify functional SLiMs in protein networks. Despite the challenges, studying vhPPIs offers valuable insights into viral pathogenesis. These interactions are transient, with few viral proteins targeting multiple host proteins to regulate their functions (De Chassey et al. 2014b). Identifying and understanding SLiMs in network biology contribute to deciphering dynamic processes in protein networks, offering clues about modes of binding and stability of interactions. In this study, our primary objectives were to evaluate the enrichment of SLiM-mediated interactions within vhPPI data. We aimed to compare the efficacy of two PPI capturing methods, namely two-hybrid and affinity purification, in investigating SLiM-mediated interactions in viruses. Furthermore, our study sought to predict novel DMIs and identify potential mimicry candidates.

## Methods

### Data acquisition and pre-processing

A comprehensive virus-host PPI (vhPPI) database, i.e., Virus-Host Network 3.0 (VirHostNet3.0) (Guirimand et al. 2015) [retrieved on: 2023-01-02] was downloaded. It was also split into well-known high throughput methods, Y2H and AP-MS, by pulling out interactions using “two hybrids” and “affinity” as keywords. vhPPIs were restricted to reviewed UniProt IDs only. Known SLiM data was downloaded from the Eukaryotic Linear Motif (ELM) database [http://www.elm.eu.org/], which contains manually curated and experimentally validated SLiM data from the literature, making it a highly reliable SLiM resource (Kumar et al. 2022). 327 ELM classes (e.g., distinct SLiMs) with experimentally validated motif instances (2278 specific protein occurrences) and associated interacting domain data (200 ELM interacting domains) were downloaded from [http://www.elm.eu.org/] on 2023-06-23.

### DMI prediction and enrichment analysis

The downloaded ELM data was used to evaluate DMI enrichment and to predict DMIs using vhPPI data. The sites for post-translational modification (MOD) and Proteolytic cleavage sites (CLV) ELM classes, which tend to be low complexity (Edwards and Palopoli 2015) were excluded for the analysis. These classes were excluded from the analysis to reduce the false discovery rate and focus on DMIs that are more likely to be true positive by reducing noise in the network. Enrichment differences were evaluated using our previously published method i.e., SLiMEnrich version 1.5.1 (Idrees et al. 2018) which explores a protein–protein interaction (PPI) network to identify pairs of proteins engaged in interaction, with the first protein either known or predicted to interact with the second protein through a DMI. Through permutation tests, it assesses the count of known/predicted DMIs against the anticipated distribution under random association of the two protein sets. This analysis yields an estimation of DMI enrichment within the dataset (Idrees et al. 2018). Enrichment was first evaluated using the ELMi-Protein strategy, which works based on known DMIs in ELM database. The DMI enrichment (E-score) was calculated as the ratio of predicted DMI (number of predicted DMIs from real PPI data) to the mean (μ) random DMI (number of predicted DMIs identified from random PPI data from permutation test) as follows.

$$Escore=\frac{{DMI}_{pred}}{{\mu DMI}_{rand}}$$

ELM instance and domain information were further incorporated to increase the size of the network and to discover new DMIs. To predict new DMIs, new SLiM instances of known ELMs were predicted using SLiMProb v2.5.1 (Davey et al. 2010) with the disordered masking feature (IUPred score >  = 0.2) (Dosztanyi et al. 2005). The predicted SLiMs were then used to predict DMIs using SLiMEnrich v1.5.1 (Idrees et al. 2018) through the ELMc-Protein (predicted SLiMs mapped to known human partner proteins via ELMs) and ELMc-Domain (predicted SLiMs mapped to Pfam-domain-containing human partner proteins) stringencies (Idrees et al. 2018). A False Discovery Rate (FDR) for individual DMIs is also estimated as the proportion of the predicted DMIs explained on average by random associations, using the mean random DMI count. Moreover, gene ontology pathway analysis of targeted human proteins was done using gProfiler (Kolberg et al. 2023) webserver and pathways with FDR < 0.05 were selected.

### *De-novo* prediction of human SLiMs mimicked by viruses.

To further explore and see if PPIs having significant DMI enrichment could be used for *de-novo* SLiM predictions, we selected a high-throughput dataset i.e., the HI-II-14 (Rolland et al. 2014) dataset which is based on a Y2H experiment, previously shown to be effective in terms of capturing DMIs (Blikstad and Ivarsson 2015). The vhPPIs (Durmus Tekir et al. 2013) and hPPIs (HI-II-14) (Rolland et al. 2014) were integrated by mapping protein partners of each viral protein in vhPPIs to their respective interactors in the human interactome. A total of 682 datasets were generated where each dataset contained a single viral protein and all the human interactors of the viral protein’s human interaction partner. FASTA sequences for each dataset were retrieved from the UniProt database and were fed to QSLiMFinder v2.30 (Palopoli et al. 2015), [ambiguity = T and cloudfix = F] with the viral protein in each dataset treated as the query sequence for the *de-novo* discovery of SLiMs. QSLiMFinder looks for any sequence motifs in this query sequence that are enriched in the rest of the dataset (e.g., viral protein motifs enriched in the human interaction partner). The P-value of each SLiM returned was estimated using default QSLiMFinder “Sig” values. Multiple testing correction for the QSLiMFinder predictions was performed by calculating the estimated approximate FDR based on the expected number of false positives, using:

$$FDR=\frac{{p}_{N}}{{n}_{p}}$$

Where p represents the returned p-value, N represents the total number of datasets, *n*_*p*_ represents number of results returned with significance p-value. Note that, unlike a traditional statistical test, a single dataset might return multiple true and/or false positive SLiM predictions.

### Simulation of poor-quality SLiM predictions.

Two control groups were generated to simulate alternative versions of the integrated dataset:

1. Random Viral Protein ("randomvProtein"): The vhPPI network underwent disruption through the shuffling of viral proteins. This resulted in the pairing of each viral protein with the human interactors of a randomly selected human protein.
2. Random Human Interactor ("randomInteractor"): Disruption of the human–human PPI network was achieved by shuffling human proteins. This led to the effective pairing of each viral protein with a randomly selected set of human proteins.

Datasets that were too small (too few unrelated protein clusters (UPC) were disregarded from the analysis. As per QSLiMFinder default settings (Palopoli et al. 2015), only datasets that had 3 + unrelated proteins (UPCs) were included in the analysis and the analysis was focused on significant datasets (QSLiMFinder default, p-value < 0.1).

### Reliability assessment of predictions using previously known ELMs

CompariMotif v3.14.1 (Edwards et al. 2008) was utilized to assess the identified motifs in comparison to previously published motifs from ELM, aiming to determine the extent of overlap and relationships between them. The classification of motifs followed the benchmarking criteria outlined in the QSLiMFinder paper (Palopoli et al. 2015). Specifically, a motif was deemed a true positive (TP) match if it satisfied the minimum match criteria of MatchIC ≥ 1.5 and normalized IC ≥ 0.5, and if the hub protein was confirmed to interact with the corresponding ELM. On the other hand, a motif was considered off-target (OT) if its pattern matched an ELM with greater stringency (MatchIC ≥ 2.5 or NormIC ≥ 1.0), but the associated ELM was not known to interact with the hub protein. Hits falling below the minimum match criteria were treated as spurious and disregarded. Motifs lacking any matches meeting the specified criteria were identified as false positive (FP) predictions if originating from control datasets, or as potential novel motifs if identified in the actual data.

## Results

**Fig. 1:**
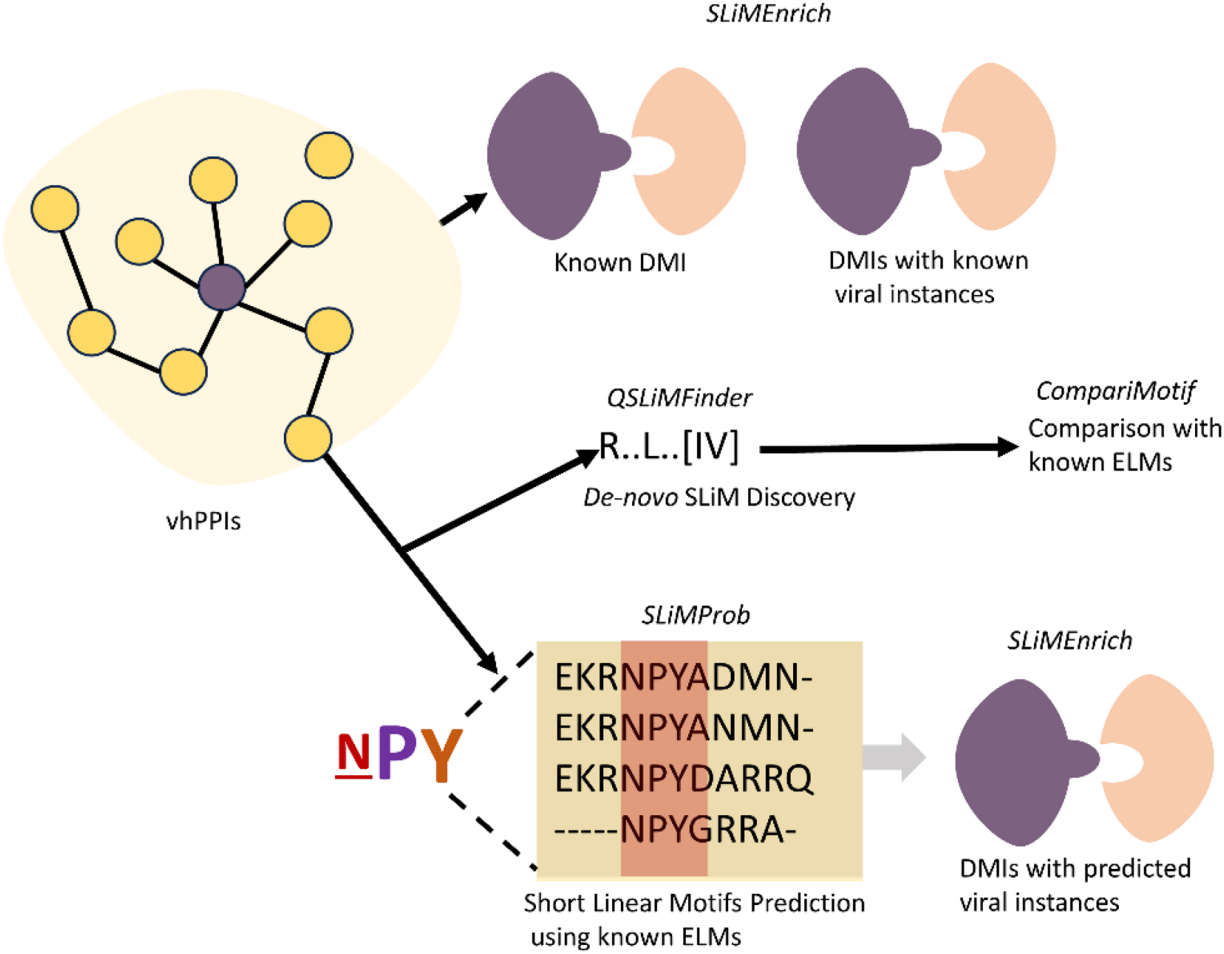
General schema of the pipeline. Firstly known interactions were recovered from virus-host interactions (vhPPIs) using ELMiProtein strategy where the vhPPIs is compared with the known interaction (virus-human protein pairs) data available in ELM database and evaluated enrichment (Kumar et al. 2022), then employed second strategy known as ELMcProtein that incorporates the Eukaryotic Linear Motif (ELM) information in the network, and finally applied ELMcDomain strategy which further expands the network through adding domain information in the network. Both ELMcProtein and ELMcDomain strategies were run with known and then predicted viral SLiMs (SLiMProb predictions). Next, *de-novo* prediction of SLiMs was done using QSLiMFinder v2.3.0 through integrating vhPPIs and a publicly available human PPI dataset

In this study, we introduce a computational pipeline to identify potential domain-motif interactions (DMIs) and potential human short linear motifs (SLiMs) mimicked by viruses. For this purpose, we used our previously published method i.e., SLiMEnrich v1.5.1. SLiMEnrich has three main strategies/stringencies (ELMiProtein, ELMcProtein and ELMcDomain) to identify DMIs from the interaction data and can work with known and predicted viral SLiMs. ELMi-Protein, characterized by the highest stringency, directly associates motif proteins with domain protein partners without utilizing motif and domain information; ELMc-Protein, with a medium stringency, establishes connections between motif classes and known domain protein partners, excluding domain information; and ELMc-Domain, is the lowest stringency, establishes connections between motif classes and known interacting domains. First, SLiM-Enrich identifies all possible DMI links and then potential DMIs are overlaid onto the PPIs to identify predicted DMIs within the PPI data (Fig. 1).

### Viral-human PPIs capture SLiM-mediated interactions.

It was of interest to examine whether vhPPI data can effectively capture DMIs and to what extent vhPPIs are enriched in capturing these interactions. For this purpose, the ELMi-Protein strategy of SLiMEnrich v1.51 was employed to identify known DMIs captured by vhPPIs available inVirHostNet3.0 (Guirimand et al. 2015). A total of 4 known DMIs were captured, with ~ 28 × enrichment compared to random (FDR < 0.05)**.** This showed that vhPPI data was indeed capturing DMIs and thus, can be used in studying molecular mimicry in viruses. We also assessed whether high-throughput screens can capture DMIs in vhPPIs. For this purpose, we filtered affinity and two-hybrid interactions from the VirHostNet3.0 dataset and looked for known DMIs. Both methods showed significant enrichment (FDR < 0.05) in terms of capturing DMIs (Table 1). However, the number of known DMIs in this data was quite low, the reason could be only a few known viral DMIs have been reported to date (~ 132 in ELM database) and this emphasizes the need to identify new DMIs that can help in studying molecular mimicry by viruses.

**Table 1:** DMI enrichment in vhPPI data available VirHostNet3.0 databases

Stringency	Method	vhPPI^1^	DMI^2^	Enrichment	FDR
ELMiProtein	All	10,736	4	28.37**	0.035
	Affinity	5,755	2	32.26**	0.031
	Y2H	1,970	1	83.3**	0.012
ELMcProtein	All	10,736	9	33.32**	0.033
	Affinity	5,755	5	44.25**	0.022
	Y2H	1,970	4	125**	0.008

** P-value < 0.001

^1^Non-redundant vhPPIs

^2^Non-redundant observed DMI

^3^FDR by SLiMEnrich

Once it was established that vhPPI data was capturing DMIs, our next aim was to use the noisier ELMc-Protein strategy (medium stringency) where known viral mimicry instances were used to predict DMIs. This was done to increase the number of DMIs and to predict new DMIs mediated by known mimicry candidates. The ELMc-Protein stringency of SLiMEnrich was used to link known viral instances to their potential human partners. A total of 9 non-redundant DMIs were predicted with an enrichment score of 33.32 (FDR < 0.05), of which only three are known in the ELM database. The high enrichment (FDR < 0.05) of additional predicted DMIs suggests their likelihood of being real (Table 2).

**Table 2:** Known and predicted DMIs using known viral instances

vProtein	Virus	Motif	hProtein
E6	Human papillomavirus type 16	LIG_PDZ_Class_1	DLG2
E6	Human papillomavirus type 16	LIG_PDZ_Class_1	MAGI1
Polyprotein	Semliki forest virus (SFV)	LIG_G3BP_FGDF_1	G3BP2
E6	Human papillomavirus type 18	LIG_PDZ_Class_1	DLG2
E6	Human papillomavirus type 18	LIG_PDZ_Class_1	MAGI1
Segment-10	Bluetongue virus 10 (isolate USA) (BTV 10)	LIG_WW_1	ITCH
Segment-10	Bluetongue virus 10 (isolate USA) (BTV 10)	LIG_PTAP_UEV_1	TSG101
gag	Human spumaretrovirus (SFVcpz (hu)) (Human foamy virus)	LIG_PTAP_UEV_1	TSG101
gag	Human immunodeficiency virus type 2 subtype A (isolate BEN) (HIV-2)	LIG_PTAP_UEV_1	TSG101

Known/Validated DMIs are shown in bold

**Fig. 2:**
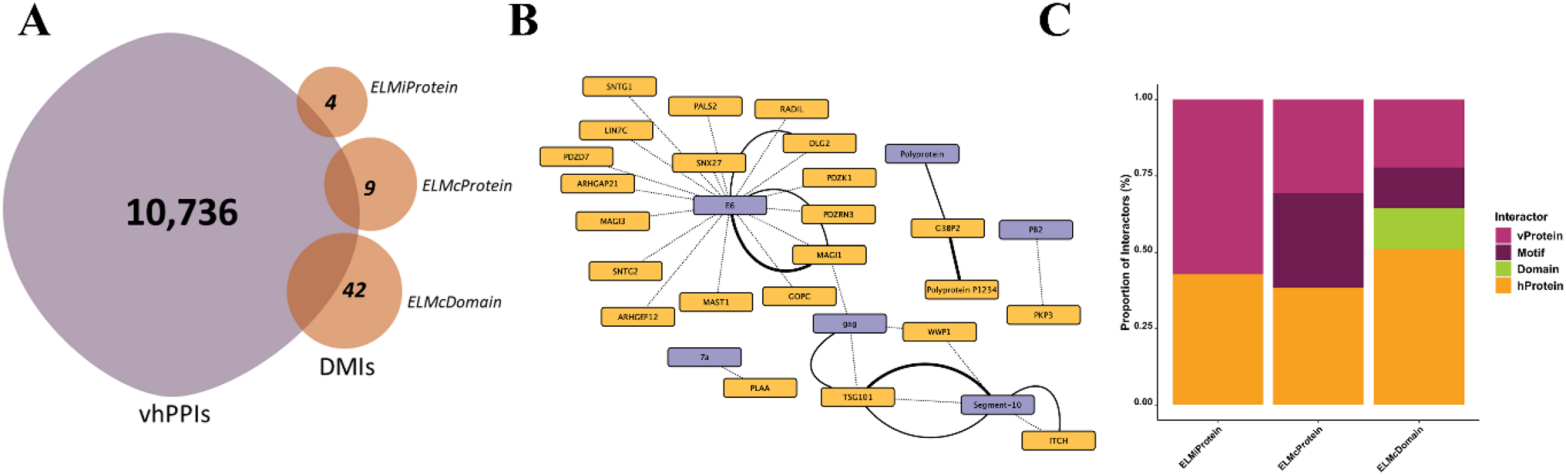
Known ELM mediated DMIs, **A** Number of DMIs predicted using known DMI/ELM information at different stringencies, **B** DMI network of known and predicted DMIs. DMIs resolved at the PPI level. Purple rectangles, viral proteins. Yellow rectangles, human proteins. Thick solid black lines, DMIs captured using ELMi-Protein strategy. Thin solid black lines, DMIs captured using ELMc-Protein strategy. Black dotted lines, DMIs captured using ELMc-Domain strategy, **C** Proportion of interacting proteins, motifs and domains captured by each stringency strategy

Ultimately, the more stringent SLiMEnrich setting (ELMcDomain) was employed to augment the predicted DMI count. In this context, established viral instances were associated with Pfam domains containing human proteins through ELMs. This led to identifying 42 non-redundant (NR) DMIs where 6 unique ELMs of 10 viral sequences interacted with 6 distinct domains of 23 host proteins (FDR = 0.0862) (Fig. 2B). The number of vhPPIs, and DMIs identified from different stringencies is shown in Fig. 2A. Gene ontology analysis revealed that viral proteins were hijacking human proteins involved in downregulation of ERBB4 signalling pathway. On a general note, the predicted DMIs had fewer viral proteins interacting with the higher number of (~ 2x) human proteins, suggesting that few viral proteins can mimic different human proteins and can hijack host cellular machinery to mediate different functions (Fig. 2C).

### DMI prediction using predicted viral instances of known ELMs.

**Fig. 3:**
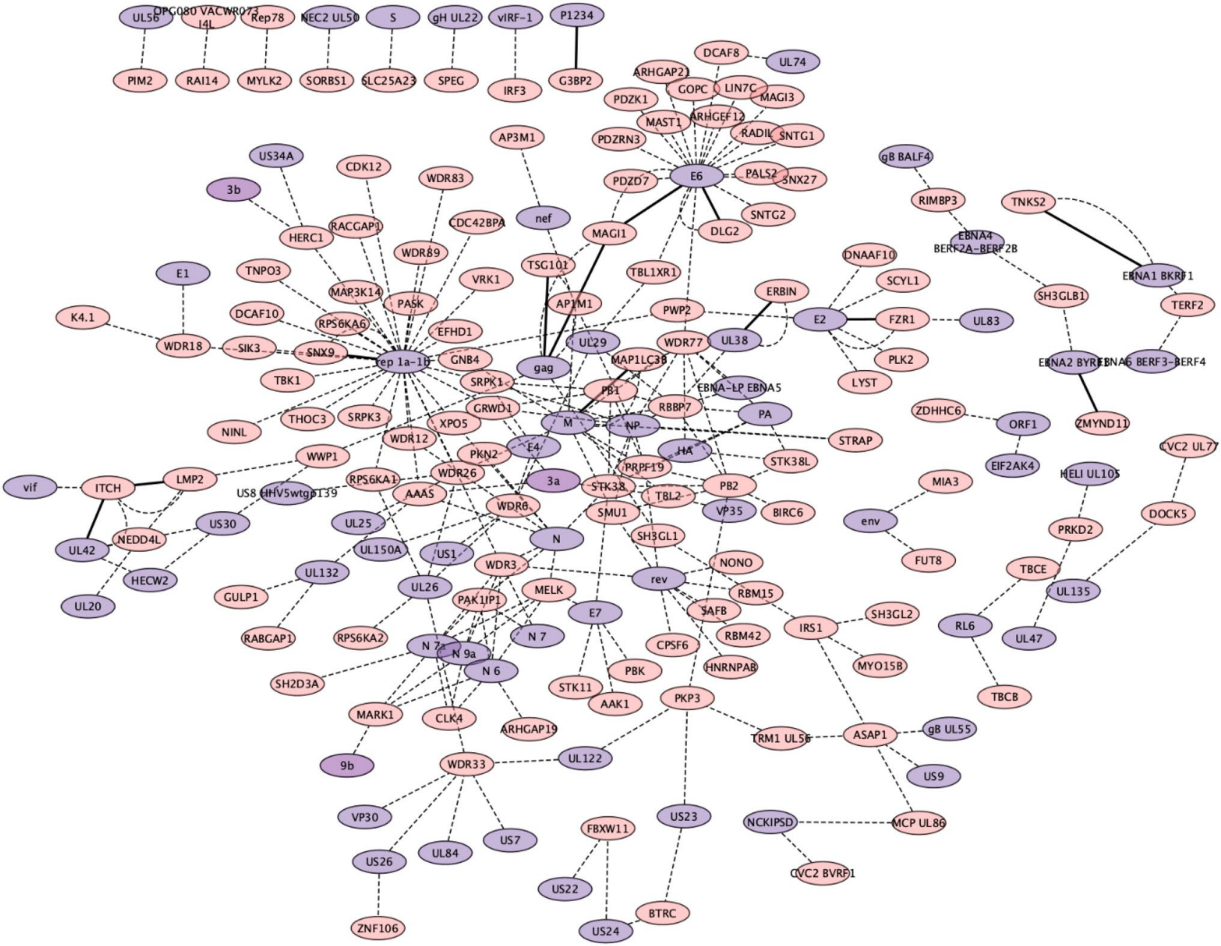
Domain-motif resolved network of predicted DMIs. Purple, viral proteins. pink, human proteins. Thick solid black lines, DMIs captured using ELMc-Protein strategy. Thin dashed black lines, DMIs captured at highest stringency i.e., ELMcDomain

To predict new candidates for molecular mimicry, SLiM instances of known ELMs were predicted in all viral proteins using SLiMProb v2.5.1 (Edwards and Palopoli 2015) with the disordered masking feature (IUPred score >  = 0.2) (Hagai et al. 2011). The predicted SLiMs were then used to predict DMIs using the ELMc-Protein strategy. A total of 23 DMIs were predicted where 11 unique motifs of 13 viral proteins were interacting with 12 host proteins with an enrichment of 4.46 (FDR = 0.22) (Table 3, Fig. 3). All identified DMIs were mostly associated with ligand (LIG) ELMs. The approximately 3% FDR for these predictions suggests a likelihood that the newly discovered DMIs could be genuine; however, it is imperative to conduct further validation. Within these DMIs, two were facilitated by LIG_WW_1, a WW domain binding motif known across various species (Traweger et al. 2002), including humans and viruses such as Human herpesvirus and Ebola virus. Among the identified DMIs, one was documented in ELM, while one was not annotated in ELM (considered predicted DMIs) (Table 3). Overall, in total, seven of these were new DMIs, not previously annotated in ELM.

**Table 3:** Predicted DMIs using predicted viral SLiMs using ELMcProtein stringency

Viral Protein	Virus	Motif	Human UniProt	Human Gene Symbol
E2	Human papillomavirus 3	DEG_APCC_DBOX_1	Q9UM11	FZR1
Vpu	Human immunodeficiency virus type 1 group M subtype B (HIV-1)	DEG_SCF_TRCP1_1	Q9UKB1	FBXW11
EBNA1 BKRF1	Epstein-Barr virus (strain B95-8) (HHV-4) (Human herpesvirus 4)	DOC_ANK_TNKS_1	Q9H2K2	TNKS2
P1234	Semliki forest virus (SFV)	LIG_G3BP_FGDF_1	Q9UN86	G3BP2
M	Influenza A virus	LIG_LIR_Gen_1	Q9GZQ8	MAP1LC3B
EBNA2 BYRF1	Epstein-Barr virus (strain AG876) (HHV-4) (Human herpesvirus 4)	LIG_MYND_1	Q15326	ZMYND11
E6	Human papillomavirus type 16	LIG_PDZ_Class_1	Q15700	DLG2
E6	Human papillomavirus type 18	LIG_PDZ_Class_1	Q15700	DLG2
E6	Human papillomavirus 33	LIG_PDZ_Class_1	Q96QZ7	MAGI1
E6	Human papillomavirus type 18	LIG_PDZ_Class_1	Q96QZ7	MAGI1
E6	Human papillomavirus type 16	LIG_PDZ_Class_1	Q96QZ7	MAGI1
E6	Human papillomavirus 31	LIG_PDZ_Class_1	Q96QZ7	MAGI1
gag	Rous sarcoma virus subgroup C (strain Prague) (RSV-Pr-C)	LIG_PDZ_Class_1	Q96QZ7	MAGI1
UL38	Human cytomegalovirus (strain Merlin) (HHV-5) (Human herpesvirus 5)	LIG_PDZ_Class_2	Q96RT1	ERBIN
gag	Human immunodeficiency virus type 1 group M subtype B (isolate HXB2) (HIV-1)	LIG_PTAP_UEV_1	Q99816	TSG101
gag	Human spumaretrovirus (SFVcpz(hu)) (Human foamy virus)	LIG_PTAP_UEV_1	Q99816	TSG101
gag	Human immunodeficiency virus type 2 subtype A (isolate BEN) (HIV-2)	LIG_PTAP_UEV_1	Q99816	TSG101
gag	Human T-cell leukemia virus 1 (isolate Caribbea HS-35 subtype A) (HTLV-1)	LIG_PTAP_UEV_1	Q99816	TSG101
ORF21	Human herpesvirus 8 (HHV-8) (Kaposi's sarcoma-associated herpesvirus)	LIG_SH3_1	Q9Y5X1	SNX9
rep 1a-1b	Human coronavirus HKU1 (isolate N1) (HCoV-HKU1)	LIG_SH3_1	Q9Y5X1	SNX9
rep 1a-1b	Human coronavirus OC43 (HCoV-OC43)	LIG_SH3_1	Q9Y5X1	SNX9
UL42	Human cytomegalovirus (strain Merlin) (HHV-5) (Human herpesvirus 5)	LIG_WW_1	Q96J02	ITCH
LMP2	Epstein-Barr virus (strain AG876) (HHV-4) (Human herpesvirus 4)	LIG_WW_1	Q96J02	ITCH

The UniProt Ids of viral and human proteins were converted to their gene names

**Fig. 4:**
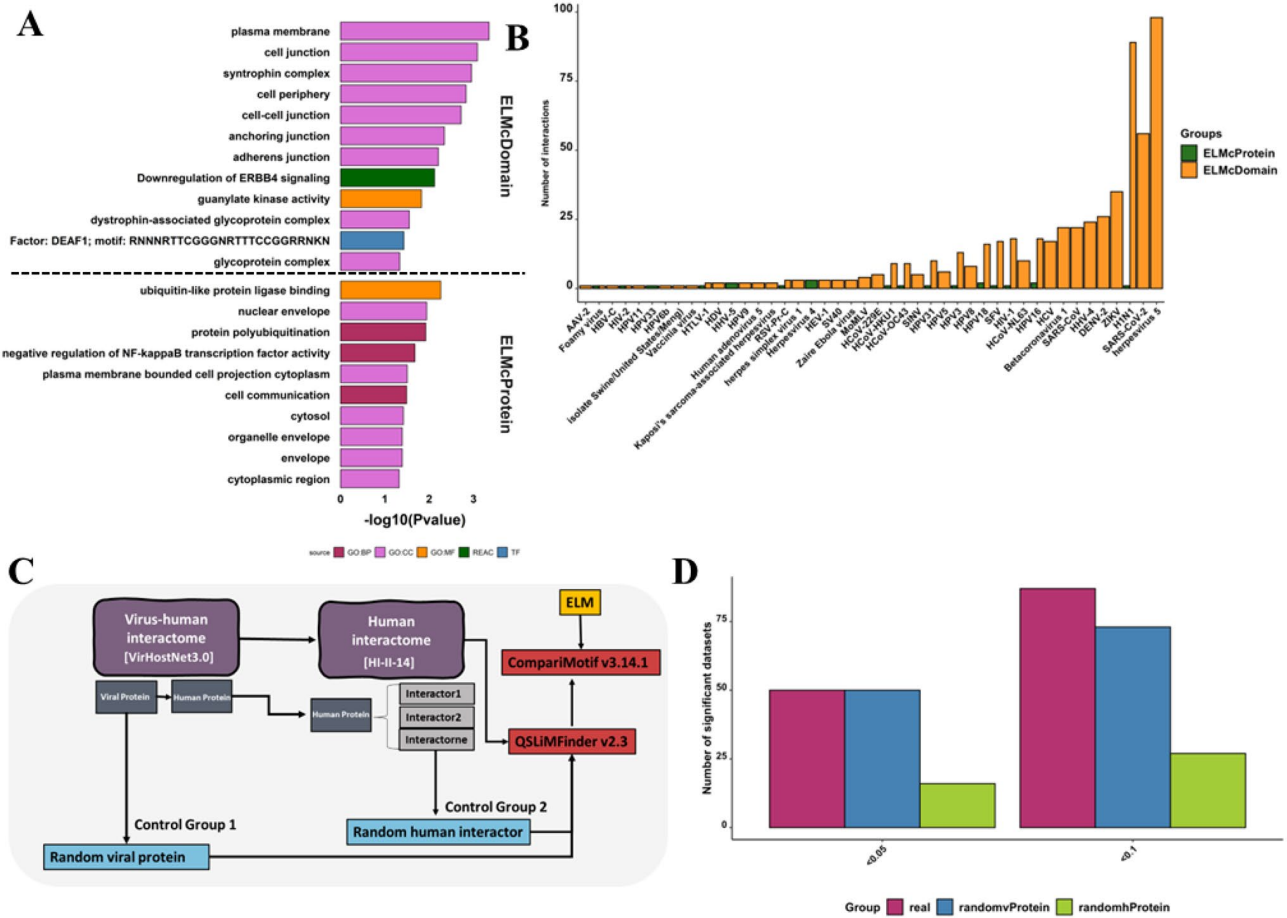
*de-novo* SLiM discovery, **A** Biological pathways targeted by predicted SLiMs, **B** Number of interactions by different viruses predicted at different stringencies, **C** Illustration of the process for *de-novo* SLiM discovery and data generation. The virus-human interactome is consolidated with the human interactome by associating each human partner protein (hProtein) with its respective human interaction partners (Interactors) in the HI-II-14 dataset. Subsequently, these human interactors are appended to the corresponding viral protein (vProtein) to create a dataset for QSLiMFinder v2.2, enabling the *de-novo* discovery of SLiMs with the vProtein serving as the query. To establish control groups, two sets were generated: the first involved randomized viral proteins, while the second involved randomized human interactor proteins. **D** Analysis of *de-novo* SLiM mimicry prediction was conducted using QSLiMFinder and CompariMotif. The graph illustrates the comparison between the number of datasets returning SLiMs and the significant p-values calculated by SLiMChance. The x-axis represents the P-value cut-off, while the y-axis depicts the number of datasets returning SLiMs with the respective cut-off P-values. Maroon bars represent real datasets, blue bars represent control group 1 with randomized viral proteins, and green bars represent control group 2 with randomized human interactors

Finally, predicted viral SLiMs were linked to human proteins via ELM-binding Pfam domains. This introduction of noise in the DMI network drastically increased the DMI number while lowering the overall enrichment. It returned 635 (393 NR) DMIs, where 44 unique motifs of 160 viral sequences interacted with 27 distinct domains of 154 host proteins, with 1.315 enrichment. However, the FDR of these predictions was quite high 0.76 (Table S1, Fig. 3). Gene ontology pathway analysis revealed that the targeted human proteins (141 unique proteins) hijacked by these viruses were involved in the downregulation of ERBB4 signalling, protein polyubiquitination, negative regulation of NF-kb activity and cell communication (FDR < 0.05). The human proteins targeted by viral SLiMs (ELMcProtein) were mainly located in envelope and cytoplasmic regions while targeted proteins identified using ELMcDomain were in cell junction and plasma membrane (Fig. 4A). Numerous DMIs were observed among Human Herpesvirus 5 (HHV-5), Severe Acute Respiratory Syndrome Coronavirus 2 (SARS-CoV-2), Influenza A Virus (H1N1), and Zika Virus based on interactions available in VirHostNet3.0 database (Fig. 4B).

### *De-novo* SLiM prediction reveals new mimicry candidates.

Since the viral-human interactome revealed significant enrichment in domain-motif interactions (DMIs), we opted to utilize it for the discovery of *de-novo* SLiMs. For each viral protein, we generated 682 datasets, each comprising a single viral protein and all human interactors of its human interaction partner. These datasets were then input into QSLiMFinder, treating viral proteins as queries to predict SLiMs. Following the SLiM discovery criteria, datasets with insufficient or excessive Unrelated Protein Clusters (UPCs) were excluded from the analysis (Palopoli et al. 2015) (Fig. 4C). Out of the initial datasets, 87 were deemed significant (p-value < 0.1), yielding 720 motifs in the real group. Considering the abundance of datasets and recognizing QSLiMFinder's lower stringency compared to SLiMFinder (Palopoli et al. 2015), we focused on results with a more stringent significance threshold (P ≤ 0.05). This refinement resulted in 50 datasets returning motifs (62 clouds, 54 motif patterns) at P ≤ 0.05. To validate the efficacy of the pipeline in *de-novo* viral SLiM mimicry discovery, we simulated two random control groups with one having randomized viral proteins and the other randomized human interactor proteins. The significant randomvProtein datasets returned 188 individual motifs (383 clouds, 52 motif patterns) at P ≤ 0.05 and 73 at P-value 0.1. On the other hand, only 16 significant random Interactor datasets returned 44 individual motifs (24 clouds, 9 motif patterns) at P ≤ 0.05 and 27 at P-value 0.1 (Fig. 4D).

Subsequently, the discovered SLiMs were compared with those available in the ELM database to identify overlapping motifs. CompariMotif v3.3.1 was employed for this purpose, considering a motif as a true positive (TP) if the hub protein was known to interact with or contain a domain interacting with the identified ELM. In the real data, 15 motifs were identified as TP, while in the randomvProtein group, there were 2 TP motifs, and in the randomInteractor group, there were none. Additionally, 378 motifs in the randomvProtein group were classified as overlapping motifs (OTs) but did not match ELM patterns based on specific criteria. No OTs were identified in the real or randomInteractor groups. Finally, a total of 979 motifs meeting certain criteria and lacking a known hub in ELM were considered new potential mimicry candidates across 15 viral species (Table S3).

## Discussion

Among the Domain-Motif Interactions (DMIs) catalogued in the ELM database (Gouw et al. 2017), only a subset is presently documented in the human interactome, underscoring the ongoing discovery of numerous DMIs within viral-human Protein–Protein Interactions (vhPPIs). So far only a few DMIs (i.e., 132 vhDMIs) have been reported in ELM, which highlights that many DMIs are yet to be discovered in vhPPIs. In our initial analysis, we identified four known DMIs: the interaction of the oncogenic E6 protein from HPV-16 with MAGI-1. The oncogenic protein E6 from HPV-16 interacts with MAGI-1 by binding its protein binding motif to the PDZ1 domain of MAGI-1, leading to MAGI-1 degradation and disruption of its role in regulating cellular signalling pathways (Araujo-Arcos et al. 2022). Interaction between Sindbis virus (SINV) polyprotein and G3BP2 was also identified, which has previously been identified in different studies (Cristea et al. 2006; Cristea et al. 2010). Third interaction was between segment 10 of Bluetongue virus 10 (BTV 10) and TSG101, which has also been demonstrated previously (Wirblich et al. 2006). Final interaction was between polyprotein of Semliki Forest virus (SFV) and G2BP2. SFV nsP3 captures G3BP, preventing the formation of stress granules on viral mRNAs (Panas et al. 2012).

Utilizing the SLiMEnrich methodology on known SLiMs within the ELM database has the potential to forecast novel mimicry candidates, revealing instances where viral proteins exploit host functions. In general, the application of this approach resulted in the rediscovery of only a small fraction of the known vhDMIs, approximately 3%, within the database. The limited representation of known vhDMIs in vhPPI datasets suggests the existence of numerous unidentified DMIs that are not present in these datasets. Upon establishing the enrichment of viral interactomes in terms of capturing DMIs, we further investigated the comparative efficacy of high-throughput methods, namely yeast two-hybrid (Y2H) and affinity purification coupled with mass spectrometry (AP-MS), in predicting DMIs. The primary goal of this analysis was to assess the proficiency of these methods in capturing DMIs from vhPPIs. Both AP-MS and Y2H vhPPI screens demonstrated substantial enrichment in terms of capturing DMIs. Given the relatively low proportion of known vhDMIs captured by vhPPIs, a medium stringency [ELMc-Protein] filtering was implemented to augment the network (Idrees 2020; Idrees and Paudel 2023b) and discover new vhDMIs. The ELMc-Protein strategy contributed additional DMIs to the results, thereby increasing the likelihood of identifying novel DMIs. Both high-throughput methods continued to exhibit significant enrichment. While our investigation suggests the potential of both Y2H and AP-MS screens in capturing vhDMIs, it is crucial to acknowledge that the current results may not conclusively establish their efficacy. These intriguing findings warrant further research and validation to bolster their reliability. Our analysis revealed instances where a few known viral proteins interacted with multiple human proteins, orchestrating the hijacking of host cellular machinery to mediate diverse functions. This phenomenon may be attributed to the viruses' compact and intricate genomes, which feature multifunctional, convergently evolved SLiMs. These SLiMs play a pivotal role in facilitating numerous DMIs, allowing viruses to effectively mimic and co-opt the host cellular machinery. In a broader context, the limited genomic resources of viruses exert significant evolutionary pressure, compelling them to mediate a specific number of DMIs with their host to sustain their life cycle. Notably, a study indicated that viral proteins, involved extensively in DMIs, possess a greater abundance of SLiMs compared to human proteins, mimicking various human proteins for their survival (Garamszegi et al. 2013).

The ELMc-Domain strategy demonstrated a modest False Discovery Rate (FDR), implying that even in the presence of noisier DMI predictions, a substantial number of them might still be genuine. In an effort to expand the pool of potential novel DMIs, the mapping stringency was further relaxed. SLiM occurrences predicted by SLiMProbv2.5.1 (Edwards and Palopoli 2015) were utilized instead of relying on known viral instances from ELM. The SLiMProb-ELMc-Protein strategy estimated approximately 21 real vhDMIs, suggesting the prediction of interactions not identified by more stringent approaches. However, the FDR for these DMIs was notably high (~ 0.2), signifying that around 20% of predicted DMIs might be false positives. Caution is advised in interpreting individual DMI predictions from this strategy. Further relaxation of the strategy to incorporate SLiMProb predictions and permit DMI predictions based on interactions between ELM classes and Pfam domain classes (i.e., SLiMProb-ELMc-Domain) substantially increased the number of predicted DMIs but drastically reduced the observed enrichment for predicted SLiM occurrences. It is important to note that, when using predicted SLiMs, the estimated false positive rate for individual DMI predictions was very high (~ 0.7). This underscores the need for caution when interpreting large-scale predictions of this nature. In summary, both strategies (ELMc-Protein and ELMc-Domain) using predicted SLiMs generated numerous DMIs, but the FDR was higher than that of known instances, necessitating careful consideration of potential false positive DMIs. This highlights the imperative to validate these new predictions rigorously, distinguishing true positives from false positives. Overall, there is a pressing need to reduce the FDR to enhance the power and reliability of such analyses. One way to improve this analysis is to have more PPI data for under-represented subtypes or categorize viral proteins based on their roles in life cycles. Implementing filtration steps for predicted DMIs to decrease the FDR could result in fewer predictions but a higher proportion of genuine ones. It was crucial, however, to first explore PPIs to answer the broader question of whether viral-human PPIs can effectively predict mimicry. Once assured that PPIs capture DMIs with significant enrichment, this knowledge could be leveraged to investigate the authenticity of predicted mimicry candidates. SLiMEnrich offers an advantage in finding DMIs by utilizing various SLiM predictions, such as SLiMFinder, which is more tolerant to noise and can be used in conjunction with SLiMEnrich to identify DMIs without significant loss of signal.

We merged the viral and human interactomes to uncover new motifs using QSLiMFinder. For control analysis, we generated two random groups to assess how dataset quality might influence motif detection. In the first control group (randomvProtein), we disrupted the viral-human interactome by shuffling viral proteins. This aimed to test the impact of randomizing viral proteins on motif search, anticipating that predicted motifs would likely be off target. In the second control group (randomInteractor), we shuffled human proteins, disrupting the hPPI network, effectively pairing each viral protein with a random set of human proteins. Since the hPPI network was disrupted, motifs needed to be more prevalent to be detected. A substantial number of datasets in each group returned motifs at SLiMChance P-value ≤ 0.1, indicating effective motif prediction. However, the number of datasets returning motifs varied among the groups. While the real group outperformed control groups at p-value < 0.1, this trend was not consistent at more stringent p-values (< 0.05). This discrepancy may suggest potential over-prediction or the dominance of false positives by QSLiMFinder. To assess prediction accuracy, we compared all returned motifs from real data with known ELMs, classifying them as true positives (TPs) or off-targets (OTs). OTs may represent generic recurring motifs or specific motifs enriched by chance or shared interactors (Edwards et al. 2012). However, OTs shouldn't strictly be considered false positives, as many are likely real SLiMs with biological significance. The best way to see how good are the predictions and how likely it is to return real motifs is to recover known/TPs from the realistic biological data (Edwards et al. 2012). A motif was considered a true positive only if the hub protein was known for interaction in ELM database. This analysis identified 15 known interaction motifs in the real group and 2 in the randomvProtein group. Given the low number of known vhDMIs in databases like ELM, the recovery of a few TPs is expected. In addition to known motifs, we identified potential mimicry candidates that could be true positives. Experimental validation of these candidates is essential for screening true positives and understanding their role in viral mimicry.

Overall, there are various strengths of this study. For instance, we proposed a computational pipeline for predicting new DMIs between viral and human proteins as well as for predicting new potential mimicry candidates. Our DMI and *de-novo* pipeline has resulted in various new DMIs and mimicry candidates. However, further experimental validation of these predictions needs to be conducted. We also present the first study to assess whether the two well-known high-throughput methods Y2H and AP-MS capture DMIs. Some caveats are also worth mentioning. This study was based on one vhPPI database, and in future it would be interesting to compare different vhPPI databases to identify more DMIs. Moreover, the *de-novo* prediction analysis relied on the integration of two datasets, namely VirHostNet3.0 and HI-II-14. The human PPI data utilized in this analysis was constrained in size compared to the comprehensive PPI databases. Consequently, enhancing the analysis by incorporating more extensive datasets holds the potential to augment predictions of new SLiMs by providing a larger pool of proteins for consideration. Additionally, previous research has demonstrated that employing masking techniques based on evolutionary conservation can enhance the sensitivity of human SLiM prediction (Davey et al. 2009). Therefore, future investigations should explore the application of such masking strategies in the context of viral mimicry to further refine and improve predictive analyses.

## Conclusion

The imitation of host protein Short Linear Motifs (SLiMs) by viruses often involves establishing low-affinity domain-motif interactions (DMIs) through these mimicked motifs. In this study, we delved into virus-host interactions as a valuable resource for capturing DMIs and observed a notable enrichment of DMIs within the vhPPI dataset. Both yeast two-hybrid (Y2H) and affinity purification coupled with mass spectrometry (AP-MS) screens demonstrated promise in identifying these interactions. Our study unveiled new host–pathogen interactions and identified new candidates for viral mimicry, warranting further exploration through both computational and experimental methodologies.

### Supplementary Information

Below is the link to the electronic supplementary material.

Supplementary file 1: (XLSX 95 KB)

## Data Availability

This article contains excerpts from Idrees' thesis published in 2020 (Idrees 2020).
